# The combination of vitamin D3 and erythropoietin alleviates acute kidney injury induced by ischemia-reperfusion via inhibiting inflammation and apoptosis

**DOI:** 10.22038/IJBMS.2020.51384.11661

**Published:** 2021-02

**Authors:** Long-yan Qin, Xin Lin, Juan Liu, Rong Dong, Jing Yuan, Yan Zha

**Affiliations:** 1 Department of Nephrology, Guizhou Provincial People’s Hospital & NHC Key Laboratory of Pulmonary Immunological Disease (Guizhou Provincial People’s Hospital), Guiyang, Guizhou, 550002, P.R. China; 2 Department of Operating Room, The First Affiliated Hospital of Guizhou University of traditional Chinese medicine, Guiyang, Guizhou, 550001, P.R. China

**Keywords:** Acute kidney injury, Apoptosis, Cholecalciferol, Erythropoietin, Inflammation, Vitamin D3

## Abstract

**Objective(s):**

Acute renal ischemia may cause acute renal dysfunction due to lack of blood supply; the manifestations are renal tubular cell apoptosis, infiltration of macrophages, and microvascular destruction. Many studies have shown that erythropoietin (EPO) and vitamin D3 (VD3) can be used to prevent or treat renal ischemia-reperfusion (I/R) injury, and VD3 may interact with EPO. In the present study, the effects of the combination of VD3 and EPO in I/R acute kidney injury were studied.

**Materials and Methods:**

Rats were divided into 5 groups: sham-operated (SHAM), AKI without treatment (AKI-control), AKI treatment with VD3(AKI+VD3), AKI treatment with EPO(AKI+EPO), AKI treatment with VD3 and EPO(AKI+VD3+EPO). The effects of the combination of VD3 and EPO on AKI were assessed by histologic, inflammation, and apoptosis studies.

**Results:**

The degree of damage in renal tissue was significantly reduced in VD3, EPO, and combined groups. Combination therapy with VD3 and EPO markedly improved Creatinine clearance rate (CCr). The combined treatment group showed the lowest F4/80+ and CD68+ expressions. The expression of Bcl-2 in the combined treatment group was higher than those in VD3 group and the EPO group, while Bax’s expression goes in the opposite direction.

**Conclusion:**

This provides further evidence that VD3 and EPO have beneficial effects in I/R injury via anti-inflammatory and anti-apoptosis pathways. The synergistic protective effect of VD3 and EPO is of profound significance in the development of new strategies for the prevention and treatment of acute kidney injury (AKI).

## Introduction

Acute kidney injury (AKI) is a common complication of critically ill patients and subsequently worsens outcomes (1, 2). The risk factors such as drugs, sepsis, and ischemia-reperfusion (I/R) may lead to AKI due to a dramatic decrease of glomerular filtration rate and acute tubular necrosis cell death (3, 4). In recent years, the global incidence of AKI is increasing with each passing year. About 13.3 million people are suffering from acute renal injury, 1.7 million people dying from acute renal injury and its complications all over the world (5). It can be seen from the recent studies, death risk of AKI patients is two times that of those not suffering from AKI, and the risk for AKI developing into chronic kidney disease (CKD) is 9 times higher (6). 

AKI may cause a series of complex processes such as adenosine triphosphate (ATP) depletion, impaired mitochondrial function, intracellular Ca^2+^, increase of oxygen free radicals, activation of the multi-enzyme system, and so on, which may lead to kidney cell damage, cell apoptosis or necrosis (7, 8). F4/80+ is a cell surface glycoprotein that changes significantly during the maturation and activation of macrophages. It can mark macrophages (9). CD68 is a specific marker for macrophages (10). The changes in the two can be observed in inflammatory response after acute renal ischemia. Bcl-2 and Bax belong to the same family, which regulates apoptosis activators by controlling the permeability of the mitochondrial membrane, such as by release of cytochrome c to affect the state of cells. They are a pair of genes opposed to each other, and their expression can be used to observe cell apoptosis after AKI (11, 12)**. **Acute renal ischemia may cause acute renal dysfunction due to lack of blood supply, the main manifestations are renal tubular cell apoptosis, infiltration of macrophages, and microvascular destruction. At present, many studies have shown that erythropoietin (EPO) is an effective drug, which can be used to prevent or treat renal I/R injury. It can protect cells by regulating different signal transduction pathways, such as by EPO and erythropoietin receptor (EPOR) activation after mitogen-activated protein kinase (MAPK) signaling pathway; EPO can also activate phosphatidylinositol 3 kinase (PI3K)/Akt, nuclear factor kappa B (NF-κB) predominate, extracellular signal-regulating kinase (ERK) signaling pathway against I/R injury, to reduce apoptosis, thus relieve renal tubular damage due to a variety of causes (13-15). In addition, because the survival of red blood cells in the body and the response to cellular stress *in vitro* are affected by exogenous vitamin D3(VD3) supplementation, normal doses of VD3 have the ability to induce ischemic tolerance, which has a protective effect on AKI. In contrast, VD3 deficiency can aggravate renal tubular injury and interstitial fibrosis after I/R injury, it can also enhance the inflammatory response (16,17). VD3 in the kidney is formed by the mitochondria of the proximal convoluted tubule, it is converted from an inactive form of 25-hydroxy VD3 into an active form 1, 25-dihydroxy vitamin D3, or ossifying triol (18). Molinari’s team and Kienreich’s team found that VD3 can reduce macrophage infiltration and reduce endothelial cell adhesion molecule expression, it can also increase nitric oxide production (19). Other studies have found that VD3 can promote the injury of muscle regeneration through the mechanisms of anti-inflammation, anti-apoptosis, and anti-fibrosis, as well as increase the immune tolerance of the body by inhibiting the production of IL-6 and up-regulating the number of regulatory T cells *in vitro* (20-22).

It has been reported that VD3 may interact with other renal hormones such as EPO and renin besides mineral metabolism. However, there is little research on the protective effects of the combined application of EPO and VD3 in AKI. Therefore, we attempt to evaluate the effects of VD3 plus EPO on the prevention of kidney I/R injury through animal experiments.

## Materials and Methods

***Experimental animals***

A total of 34 adult male Sprague Dawley (SD) rats, weighing 250–280 g, were used in this study, which were purchased from Guizhou Medical University (Guiyang). The reported experiment was conducted in accordance with the standards of the eighth edition of the Laboratory Animal Care and Use Guidelines. The experimental protocol was approved by the Animal Committee of Guizhou Provincial People’s Hospital (Guiyang, China).

***Experimental drug***

Peanut oil is produced by China Luhua Luxiang Peanut Oil Co., Ltd., and Alfa Calcium Oxide Soft Capsule (0.25 ug×20 capsules) is produced by Dalian Tianyu Aosen Pharmaceutical Co., Ltd., China. Recombinant human erythropoietin injection (3000 IU/0.6 ml) / Branch × 5) is produced by Beijing Sihuan Bio-Pharmaceutical Co., Ltd., China.

***Animal model***

**Figure 1:**
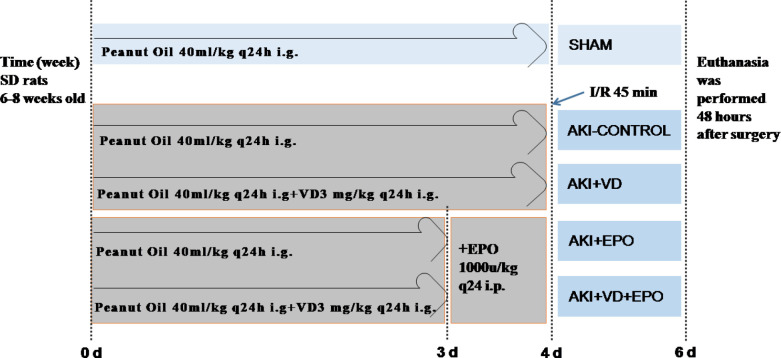
Experimental grouping and flow-chart

In this study, rats were fed normal feed and water according to the above guidelines and placed in Guizhou Provincial People’s Hospital. Thirty four SD rats (250–280 g) were randomly divided into 5 groups: sham-operated (SHAM; n=5), AKI without treatment (AKI-CONTROL; n=6), AKI treatment with VD3(AKI+VD; n=7), AKI treatment with EPO(AKI+EPO; n=8), AKI treatment with VD3 and EPO(AKI+VD+EPO; n=8) (Figure 1). All rats in the I/R group underwent the same surgery. The rats were anesthetized with chloral hydrate (450 mg/kg) and, if necessary, supplemented with the same anesthetic (25% of the initial dose) to maintain anesthesia (23), and the body temperature was kept constant at 37 °C with a thermostatic pad during the operation. The abdominal organs were exposed through the midline of the abdominal cavity, the position of the kidneys on both sides was observed, and the renal arteries on both sides were carefully separated. The bilateral renal artery was clamped with a non-traumatic vascular clamp to block blood supply for 45 min. Blood perfusion is judged by changes in the color of the kidney surface. After removing the blood vessel clip and another 5 min of observation, the color change to red blood was determined. After the blood is reconstituted, 0.5 ml of physiological saline was intraperitoneally injected to suture the muscle and skin. These rats were allowed to survive for 48 hr. Sham surgery was performed in a similar manner except that the renal blood vessels were not clipped. After 48 hr, according to the AVMA guidelines for the euthanasia of animals (2013 edition, available at ww.avma.org/KB/Policies/Documents/euthanasia.pdf. accessed Feb 7, 2019), the rats were euthanized by cervical dislocation. Before cervical dislocation, the rats were anesthetized with chloral hydrate (3 times the initial dose). During the experiment, the rats would be euthanized if they suffered from weight loss (15% of the initial weight), loss of appetite, weakness, nonhealing wounds, or other conditions determined by professional animal keepers. The rat’s cardiac arrest, pupil dilation, and respiratory arrest were used to confirm the rat’s death. Urine was collected 24 hr before sacrifice. Abdominal aortic blood was taken 7 ml/piece, centrifuged for 5 min, 3000 rpm/min, and the supernatant was taken for determination of renal biochemical indicators, and serum urea and creatinine levels were used as indicators of renal dysfunction. The kidneys of the rats were taken out, cut into two halves, fixed in 10% formalin solution for four hours, and dehydrated and embedded in paraffin.

***Assessment of renal biochemical markers***

Creatinine and serum urea concentrations were determined using a creatinine assay kit and serum urea assay kit, respectively, according to the protocols provided by the manufacturers.

***Histopathology***

The wax block was cut into 2 um sections for histopathological staining. Tissue damage was observed by Hematoxylin-Eosin (HE) staining and Periodic Acid-Schiff stain (PAS). It was checked whether the renal tissue has diffuse mononuclear-macrophage infiltration, glomerular cystic enlargement, renal tubular dilatation at the junction of the renal cortex, tubular degeneration of small cell epithelial cells, vacuolar degeneration, or brush-like detachment. In severe cases, the basal membrane of renal tubules is exposed, and there are a lot of eosinophilic substances in the tubules, and even the protein casts cause the occlusion of the tubules. A semi-quantitative score of renal tubular necrosis was performed. The scoring standard was that each slice was randomly selected under a microscope with 10 fields of view and a magnification of 200 times. The pathological score was 0–5 points. 0 is for no damage, 1 point for damage area <10%, 2 points for damage area >10% but <25%, 3 points for damage area>25% but <50%, 4 points for damage area>50% but <75%, 5 points if damage area >75%.

***Immunohistochemistry***

The embedded wax blocks were cut into 4 um sections for immunohistochemical staining. After dewaxing the sections and rehydration, 30% hydrogen peroxide (diluted with 3% hydrogen peroxide) was used to reduce non-specific staining by endogenous peroxidase. The sections were exposed to high pressure in 0.01 mmol of citrate buffer (pH 6.0) to expose intracellular epitopes. They were incubated overnight in a 4 °C refrigerator with primary antibody against the following proteins: Anti-Vitamin D Receptor antibody (CAT number: ab3508; ABCAM, Cambridge, UK; rabbit polyclonal antibody; 1:3000), Anti-F4/80 antibody (CAT) No.: ab16911; ABCAM, Cambridge, UK; rat monoclonal antibody; 1:100), Anti-CD68 antibody (CAT number: ab201340; ABCAM, Cambridge, UK; mouse monoclonal antibody; 1:200), Anti-Bax antibody (CAT number: ab69643; ABCAM, Cambridge, UK; rabbit polyclonal antibody; 1:50), Anti-Bcl-2 antibody (CAT number: ab194583; ABCAM, Cambridge, UK; rabbit polyclonal antibody; 1:100). Anti-F4/80 antibody and Anti-CD68 antibody were used as secondary antibodies in the mouse two-step kit (Nakasu Jinqiao, Beijing), Anti-Vitamin D Receptor antibody, Anti-Bax antibody, Anti-Bcl 2 antibody rabbit. The gait kit (Zhongshan Jinqiao, Beijing) was used as a secondary antibody for 30 min after DAB reagent (Zhongshan Jinqiao, Beijing). Negative control samples were treated with PBS (Nakasugi Jinqiao, Beijing). Finally, the nuclei were stained with fine hematoxylin (Solebao, Beijing) and observed under a light microscope.

## Results

***The changes in renal function in AKI rat models***

**Figure 2:**
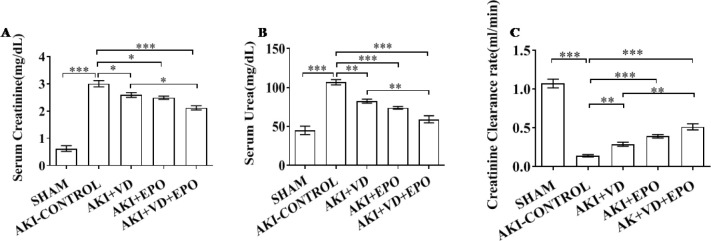
Metabolic parameters changes in AKI rat

Notes: (A) Serum creatinine level, (B) Serum urea level, and (C)Creatinine clearance rate; ***P<0.001, **P<0.01, *P<0.05. one-way ANOVA followed by Tukey’s multiple comparison test

AKI: Acute kidney injury; VD: Vitamin D; EPO: Erythropoietin

We measured serum creatinine, serum urea, and creatinine clearance rate (CCr) in the model rats. After 45 mins of I/R, blood creatinine and serum urea levels in the AKI-CONTROL group were significantly higher than those in the SHAM group but decreased CCr in response to AKI-CONTROL rats was observed (*P*<0.001). It was clear that the serum creatinine and serum urea levels were declined after pretreatment with drugs, especially after combined treatment, and CCr was significantly restored (*P*<0.001) (Figure 2). Compared with the AKI-CONTROL group, a tendency towards lower serum creatinine was seen in the VD3 group and EPO group pretreated rats (*P*<0.05), and there was no significance between the two drugs. However, combined use of VD3 and EPO is more effective than VD3 alone in the treatment of I/R injury (*P*<0.05) (Figure 2A). After drug intervention, CCr showed a trend of improvement, especially in the combination therapy group (*P*<0.001) (Figure 2C).

***The histological manifestations of kidney in AKI rat models with different interventions***

**Figure 3:**
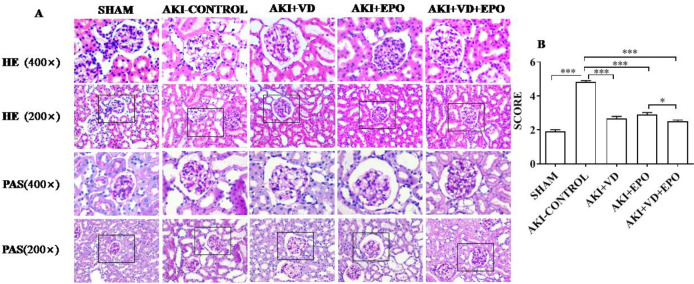
Evaluation of degree of acute kidney injury on different treatments

Notes: (A) Representative kidney histology of Hematoxylin-Eosin stain and Periodic Acid-Schiff stain (magnification 400×, 200×).Data analysis of (B) Semi-quantitative assessment of the histological lesions based on tubular necrosis; ***P<0.001, *P<0.05; one-way ANOVA followed by Tukey’s multiple comparison test

HE: Hematoxylin-eosin stain; PAS:Periodic Acid-Schiff stain; AKI: Acute kidney injury; VD: Vitamin D; EPO: Erythropoietin

The histopathological feature changes were particularly significant at the point of 48 hr after I/R operation. In order to detect AKI lesions, HE staining and PAS staining were performed (Figure 3A). Obviously, AKI abounds in diffuse renal tissues manifests itself as mononuclear macrophage infiltration, tissue interstitial edema, bowman’s capsule cavity increase, part of the tubular epithelial cells, granular degeneration, vacuoles degeneration, renal tubular basement membrane nudity, renal tubular cavity with a large number of red dye material, protein type tube, and part of the renal tubular cavity cracked. These pathological changes were alleviated in the drug intervention groups. Although some glomerular cystic lumen dilated and renal tubular epithelial cells were degenerative and exfoliated, protein-tubule and erythrocyte tubules, tube wall ruptures were rare, and basement membrane was relatively intact. According to the semi-quantitative renal tubular score (Figure 3B), compared with the SHAM group, the damage of renal tissue in the AKI-control group was significantly increased (*P*<0.001), and it was significantly relieved after VD3, EPO, and combined interventions (*P*<0.001). It is worth noting that the score of renal tissue damage after combined administration is lower than that after EPO alone (*P*<0.05).

***Expression of VDR in renal tissue of AKI rats***

**Figure 4:**
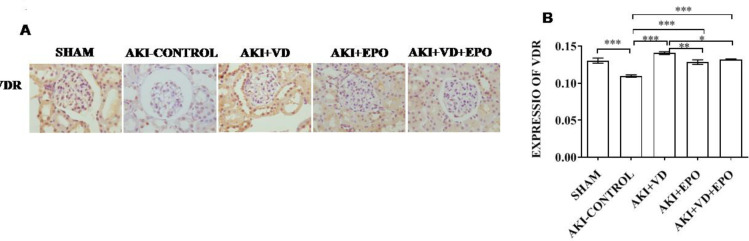
VDR expression levels in AKI rat kidneys

Notes: (A) Expression of VDR by immunohistochemistry (magnification 400×). Data analysis of (B)expression of VDR; ***P<0.001, **P<0.01, *P<0.05; one-way ANOVA followed by Tukey’s multiple comparison test

AKI: Acute kidney injury; VD: Vitamin D; EPO: Erythropoietin; VDR: Vitamin D receptor

We used immunohistochemical staining to determine vitamin D receptor (VDR) expression levels because VDR mediates the protective role of vitamin D in the kidney. VDR is mainly expressed in proximal and distal renal tubular epithelial cells but also in collecting tubular epithelial cells (Figure 4A). AKI resulted in a significant reduction in activating vitamin D receptors (*P*<0.001). After drug interventions, the expression of VDR in rat kidney tissue was significantly increased, and the effect was most prominent after VD3 treatment. No significant difference in the EPO group and combination group on VDR expression was demonstrated in the present experiment, but a downtrend was seen in these two groups compared with the VD3 group (Figure 4B).

***Different factors intervene in the inflammation of kidney tissue in AKI***

**Figure 5:**
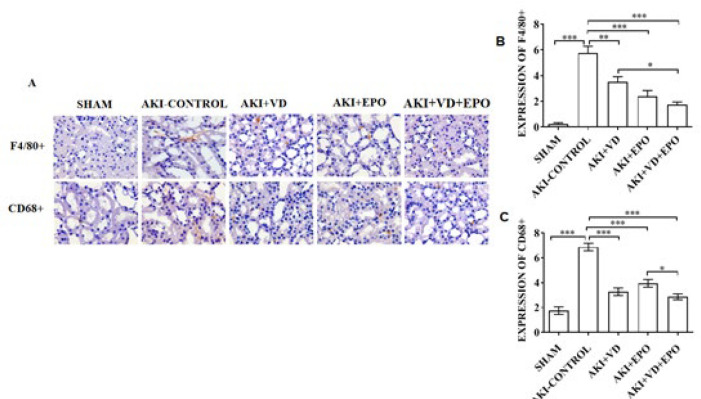
Evaluation of degree of inflammation in AKI rat kidneys

Notes: (A)F4/80+ cell, CD68+ staining in kidney sections of different groups by immunohistochemistry (magnification 400×). Inflammation changed molecularly in kidney tissue: (B) Data analysis of F4/80+, (C) Data analysis of CD68+; ***P<0.001, **P<0.01, *P<0.05; one-way ANOVA followed by Tukey’s multiple comparison test

AKI: Acute kidney injury; VD: Vitamin D; EPO: Erythropoietin

Immunohistochemical staining was used to detect the expression levels of renal inflammatory markers (F4/80+ and CD68+) in the model rats (Figure 5A). The expression of F4/80+ and CD68+ was normal in the SHAM group but significantly increased after I/R injury (*P*<0.001). The expression of the two inflammatory markers was significantly reduced after the drug intervention. F4/80+ had a downward trend after using VD3 pretreatment (*P*<0.01), and it decreased significantly after the combined application compared with VD3 used alone (*P*<0.001) (Figure 5B). The expressions of CD68+ were significantly reduced in all three intervention groups (*P*<0.001) (Figure 5C). Compared with the AKI-CONTOL group, the combined treatment group showed the lowest F4/80+ and CD68+ expressions (*P*<0.05).

***Different factors interfere with the apoptosis of kidney tissue in AKI***

**Figure 6:**
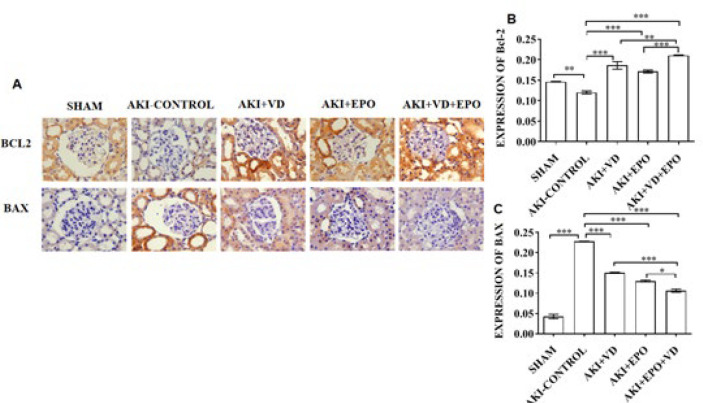
Evaluation of degree of apoptosis in AKI rat kidneys

Notes: (A)Bcl2, BAX staining in kidney sections of different groups by immunohistochemistry (magnification 400×). Apoptosis changed molecularly in kidney tissue. (B)Data analysis of Bcl2, (C) Data analysis of BAX; ***P<0.001, **P<0.01, *P<0.05; one-way ANOVA followed by Tukey’s multiple comparison test

AKI: Acute kidney injury; VD: Vitamin D; EPO: Erythropoietin

In order to further clarify the degree of apoptosis in rat kidney cells, we selectively tested the apoptosis-related proteins Bcl-2 and Bax (Figure 6). The results showed that AKI could induce decreased expression of anti-apoptotic protein Bcl-2 and increased expression of pro-apoptotic protein Bax. Bax staining is prominent in the damaged renal tubular epithelial cells (Figure 6A). Compared with the AKI-CONTROL group, the expression of protein Bcl-2 increased in the sham and all three intervention groups, and we clearly found that the increase of Bcl-2 in the administration group even exceeded that in the sham group, while Bcl-2 in the combined treatment group was the highest. The expression of protein Bax was significantly increased in both operation groups (*P*<0.001). However, we found that the expression of Bax decreased significantly after administration compared with the AKI-CONTROL group. Among them, Bax expression decreased most significantly after combination therapy compared with the VD3 group and EPO alone group (Figure 6C).

## Discussion

AKI, especially caused by I/R, increases the morbidity and mortality of hospital patients and brings a heavy burden to patients and health services. In some developing states, the treatment of AKI patients has become a huge medical burden for the families and society (1, 24-27). However, unlike other non-communicable diseases, AKI is preventable. In the past 10 years, many people have conducted extensive research and made some substantial progress in the field of AKI, but its incidence has not changed (28). Therefore, research on potential renal protective therapy to improve or prevent AKI treatment is still in progress.

According to the results of our study, the kidney was significantly damaged after I/R, serum creatinine and blood urea nitrogen increased, and renal histopathological characteristics were obvious. AKI induced by renal I/R is manifested as cell necrosis, apoptosis, and immune cell infiltration at the cellular level. To date, prophylactic use of EPO or VD3 has been shown to be one of the most effective approaches for AKI in animal models and some anthropological studies (29-31). 1, 25-dihydroxy vitamin D3, a regulator that promotes the absorption and maintenance of serum calcium, also has been reported to have multiple effects on kidney disease. After I/R injury, vitamin D deficiency can worsen renal tubular interstitial injury and renal capillary remodeling factor (32, 33). The role of EPO is mainly to promote erythropoiesis, and countless studies have shown that the EPO intervention before an ischemic attack can significantly inhibit the damage to renal tubules (34). Meanwhile, EPO can improve erythrocyte permeability during ischemia and hypoxia. It has been reported that the decrease of endogenous EPO will lead to irreversible apoptosis of cells, because EPO production has an incubation period, which is too late to protect tissues, and I/R produces a large number of proinflammatory cytokines, which will inhibit the production of EPO. Therefore, exogenous EPO supplementation is particularly critical (35, 36).

 VD3 pretreatment can reduce renal oxidative stress, and may affect the inflammatory cytokines interleukin-2 (IL-2), interleukin-6 (IL-6), interleukin-12 (IL-12), interleukin-18 (IL-18), and monocyte chemotactic protein 1 (MCP1) which is involved in the renal immune inflammation. Meanwhile, this procedure may induce the interaction of VDR expression with the NF-κB p50 subunit, thus playing an anti-inflammatory role in macrophages (37-39). Wang X *et al*. believed that VD pretreatment could inhibit apoptosis, down-regulate the expression rate of Bax, and increase the expression of Bcl-2, to protect renal tissue (40).

EPO exerts a certain anti-inflammatory effect by inhibiting the infiltration of mononuclear macrophages and the increase of the interstitial area of small cells. As our study shows, EPO can increase the anti-apoptotic Bcl-2 protein in the Bcl-2 family and reduce the pro-apoptotic protein Bax, to maintain the activity and integrity of mitochondria, inhibit the apoptosis of renal tubular cells, and decrease renal injury. These results were consistent with other studies, suggesting that EPO may play an anti-apoptotic role in renal cells through multiple signaling pathways (41-43). Therefore, it is reasonable to assume that both VD3 and EPO have protective effects on acute renal injury induced by I/R. So, we detected and compared the indicators of biochemical, renal histopathological features, inflammation, and apoptosis, after pretreatment with VD3 or EPO, I/R kidney damage did tend to alleviation. We can infer that VD3 and EPO pretreatment do have a protective effect on renal I/R injury.

Although animal models have been used in many studies to explore the protective effect of VD3 on AKI, most of them are clinically used for the treatment of chronic kidney diseases such as secondary hyperparathyroidism in chronic kidney failure. However, the clinical application of EPO has not achieved satisfactory efficacy. The incidence and cure rate of AKI is still very low. Dora Ben Alon *et al*. found *in vitro* that vitamin D plays a major role in the production of normal red blood cells (44). VD3 stimulates the proliferation of hematopoietic cells in conjunction with EPO, and EPO receptors were up-regulated because of VD3 treatment (45, 46). Immune cells express the vitamin D receptor (VDR) which in turn is involved in the modulation of innate and adaptive immunity. EPO cells also express calcitriol receptors, VDR activation inhibits the expression of inflammatory cytokines in stromal and accessory cells, it also upregulates the release of interleukin-10 (IL-10) by lymphocytes (46). However, it is difficult to know whether the combination of VD3 and EPO is more protective against acute renal injury induced by I/R than using VD3 or EPO alone. 

In our model, VDR expression was significantly increased in intervention groups. Compared with the AKI-control group, the combined treatment group showed the lowest F4/80+ and CD68+ expressions. In the aspect of apoptosis, Combination therapy significantly reduces the number of apoptosis cells in AKI. It might be that VD3 increases the effect of EPO, causing a cascade reaction on AKI.

## Conclusion

The data in this study suggest that VD3 and EPO have a great preventive effect on renal damage caused by I/R. These beneficial effects are closely related to anti-inflammatory and anti-apoptosis pathways. There are numerous studies on EPO and VD3 prevention of I/R injury, as far as we know, this study is the first to report the synergistic protective effect of VD3 and EPO on I/R injury, which is of profound significance in the development of new strategies for the prevention and treatment of renal diseases.
